# Larval cestodes infecting commercial fish of Alexandria coast along the Mediterranean Sea: morphology and phylogeny

**DOI:** 10.1590/S1984-29612022030

**Published:** 2022-06-06

**Authors:** Kareem Morsy, Saad Bin Dajem, Mohammed Al-Kahtani, Attalla El-kott, Essam Ibrahim, Hamida Hamdi, Amin Al-Doaiss, Mohamed Abumandour, Haitham El-Mekkawy, Diaa Massoud, Asmaa Adel, Shams Abd El-Kareem

**Affiliations:** 1 Biology Department, College of Science, King Khalid University, Abha, Saudi Arabia; 2 Zoology Department, Faculty of Science, Cairo University, Cairo, Egypt; 3 Zoology Department, Faculty of Science, Damanhour University, Damanhour, Egypt; 4 Blood Products Quality Control and Research Department, National Organization for Research and Control of Biologicals, Cairo, Egypt; 5 Research Center for Advanced Materials Science (RCAMS), King Khalid University,Abha, Saudia Arabia; 6 Biology Department, Faculty of Science, Taif University, Taif, Saudi Arabia; 7 Anatomy and Histology Department, Faculty of Medicine, Sana'a University, Sana’a, Republic of Yemen; 8 Anatomy and Embryology, Faculty of Veterinary Medicine, Alexandria University, Alexandria, Egypt; 9 Biology Department, College of Science, Jouf University, Sakaka, Al-Jouf, Saudi Arabia; 10 Zoology Department, Faculty of Science, South Valley University, Qena, Egypt; 11 Zoology Department, Faculty of Science, Minia University, Minia, Egypt

**Keywords:** Trypanorhyncha, Callitetrarhynchus, Protogrillotia, Grillotia, marine fish, phylogeny, Trypanorhyncha, Callitetrarhynchus, Protogrillotia, Grillotia, peixe marinho, filogenia

## Abstract

Members of the order Trypanorhyncha are cestode parasites that are frequently found infecting the muscles of several marine fish species, affecting fish health and resulting in consumers’ rejection. Seventy–five specimens of marine fish were freshly caught from boat landing sites at the Alexandria coast along the Mediterranean Sea in Egypt, including two Carangids, the greater amberjack *Seriola dumerili* and the gulley jack *Pseudocarans dentex*; two Serranids, the Haifa grouper *Epinephelus haifensis* and the mottled grouper *Mycteroperca rubra*. Forty-five fish were infected; the infection was recorded as blastocysts embedded in fish flesh. Blastocysts were isolated and ruptured; the generated plerocerci were described morphologically, where, four different species were recovered; *Callitetrarhynchus gracilis*, *Callitetrarhynchus speciosus*, *Protogrillotia zerbiae*, and *Grillotia brayi*. The taxonomic position of these parasites was justified by multiple-sequence alignment and a phylogenetic tree was constructed following maximum likelihood analysis of the 18s rRNA sequences of the recovered worms. The accession numbers MN625168, MN625169, MN611431and MN611432 were respectively assigned to the recovered parasites. The results obtained from the molecular analyses confirmed the morphological records of the recovered parasites. Since metacestodes are found in the musculature of infected fish specimens, it is necessary to remove these areas in the commercialization of fish.

## Resumo

Os membros da ordem Trypanorhyncha são cestoides parasitos, frequentemente encontrados infectando os músculos de várias espécies de peixes marinhos, afetando a saúde dos peixes e resultando na rejeição por parte dos consumidores. Setenta e cinco espécimes de peixe marinho foram capturados, recentemente, nos locais de desembarque em barcos na costa de Alexandria, ao longo do Mar Mediterrâneo no Egito, incluindo dois Carangídeos, o maior "amberjack" *Seriola dumerili* e o "gulley jack" *Pseudocarans dentex*; dois Serranídeos, a garoupa Haifa *Epinephelus haifensis* e a garoupa mosqueada *Mycteroperca rubra*. Quarenta e cinco peixes foram infectados e a infecção foi registada como blastocistos embutidos na carne do peixe. Os blastocistos foram isolados e rompidos e os pleurocistos gerados foram descritos morfologicamente, nos quais, quatro espécies diferentes foram recuperadas: *Callitetrarhynchus gracilis*, *Callitetrarhynchus speciosus*, *Protogrillotia zerbiae*, e *Grillotia brayi*. A posição taxonômica destes parasitos foi justificada pelo alinhamento de sequências múltiplas e foi construída uma árvore filogenética após a análise de máxima probabilidade das sequências de rRNA dos anos 18 dos vermes recuperados. Os números de adesão MN625168, MN625169, MN611431 e MN611432 foram respectivamente atribuídos aos parasitos recuperados. Os resultados obtidos a partir das análises moleculares confirmaram os registos morfológicos dos parasitos recuperados. Uma vez que se encontram metacestodes na musculatura de espécimes de peixe infectados, é necessário remover estas áreas na comercialização de peixe.

## Introduction

Members of the order Trypanorhyncha (Diesing, 1863) represent parasitic cestodes of fish and sea invertebrates; adults infect the stomach and intestines of sharks and rays as definitive hosts, while the larval stages are found in the musculature and coelomatic cavity of teleosteans as intermediate hosts (Campbell & Beveridge, 1994; Palm, 2004; Morsy et al., 2013; Santoro et al., 2020). Detection of these parasites among infected fish poses marketing problems (Morsy et al., 2013). Humans can be accidentally infected by larvae of Trypanorhyncha after ingesting raw fish meat which, in most cases, leads to allergic reactions. Further, the presence of larvae in the fish musculature may release toxins that affect humans (Caira & Jensen, 2017). Previous reports have concluded that experimental inoculation of Trypanorhyncha species extracts are responsible for immune responses in mice, indicating the possibility of allergic reactions in humans (Vázquez-López et al., 2001; Gòmez-Morales et al., 2008; Al Quraishy et al., 2019). Despite the worldwide distribution of these parasites in commercial fishes, and the great diversity of their species, trypanorhynchids are still a relatively poorly studied group (Palm, 2004; Menezes et al., 2018). Only a few life cycles are completely known, but those that involve several intermediate hosts before the final infestation of sharks are still missed. Few reports have been published on these parasites, likely due to the challenges associated with classification (Menezes et al., 2018). Trypanorhynchid cestodes are characterized by the presence of two or four bothria and a tentacular apparatus, which consists of tentachular sheaths with tentacles that bear numerous hooks. The hooks originate at the anterior extremity of bulbs and extend in a spiral anteriorly toward the scolex (Dollfus, 1942; Richmond & Caira, 1991; Campbell & Beveridge, 1994; Palm, 1995, 1997). Taxonomists originally identified the species of a larva in an invertebrate or teleost intermediate host based on the shape of the scolex, number of bothria, tentacular armature (Palm & Caira, 2008), zoogeographical distribution (Palm, 2004; Palm et al., 2007), and parasite evolution (Palm & Klimpel, 2007; Palm et al., 2009) as the most important morphological features of the trypanorhynchid taxonomy. Also, the taxonomy of trypanorhynchids can be justified by molecular analysis of the 18S rRNA gene which is a common molecular marker for biodiversity studies since it is highly conserved intra-species and assist in species-level analyses. In the present study, a parasitological survey for trypanorhynchid metacestodes infecting marine fish of the Mediterranean Sea at Alexandria coast in Egypt were carried out, where, the taxonomic status of the isolated parasites was determined based on both morphological characterization and the molecular analysis of the parasites’ 18s rRNA.

## Materials and Methods

A total of 75 specimens of marine fish were freshly caught throughout 2020 from boat landing sites at the Alexandria coasts along the Mediterranean Sea, Egypt. These included the greater amberjack *Seriola dumerili*Risso (1810) (F: Carangidae, no. 15), the gulley jack *Pseudocarans dentex* Bloch and Schneider (1801) (F: Carangidae, no. 20), the Haifa grouper *Epinephelus haifensis* Ben**-**Tuvia (1953) (F: Serranidae, no. 17), and the mottled grouper *Mycteroperca rubra* Bloch (1793) (F: Serranidae, no. 23). Fish specimens were transported to the laboratory and were morphologically identified, according to the methods of Kvach et al. (2018).

### Morphology

After dissection, blastocysts were isolated in an isotonic saline solution (7%) in a Petri dish, where they were ruptured to release the coiled larvae that were left to relax between two slides within hot 10% formalin as a fixative. The fixed worms were washed with distilled water to remove the excess fixative. The worms were stained using acetic acid alum carmine (Carleton, 1976). Dehydration was achieved using an ascending series of ethyl alcohol, cleared in clove oil and xylene, and then the worms were permanently mounted in Canada balsam (Ergens, 1969). The worms were subsequently examined and photographed using a BX53 microscope (Olympus Corporation, Toyko, Japan) and drawn using a camera lucida. Nomenclature of the different body parts followed the convention published by Jones et al. (2004) for trypanorhynchids. Measurements were given in millimeters (mm) and were reported as means and ranges in parentheses. To study the surface ultrastructure of worms by scanning electron microscopy (SEM), the worms were fixed in buffered glutaraldehyde (3%, pH 7.3, 3 hours), washed in the same buffer, and post–fixed in osmium tetroxide (4 hours) according to the instructions detailed by Madden & Tromba (1976). The worms were dehydrated in acetone solution, dried in a BOMER-900 drier (Leica Microsystems, Wetzlar, Germany), mounted on an aluminum stub, coated with gold palladium in a JEOL JEC-3000FC, and then examined with a JSM-6060LV microscope (JEOL, Tokyo, Japan) at 10 kV.

### DNA extraction, PCR, and sequencing

Genomic DNA (gDNA) was extracted from the preserved samples in 70% ethanol using a DNeasy tissue kit (Qiagen, Hilden, Germany) following the manufacturer’s instructions. Polymerase chain reaction (PCR) amplification of partial 18s ribosomal RNA sequences was carried out on an MJ Research PTC-150 thermocycler (Marshall Scientific, Hampton, NH, USA) using the universal primers 1F 5′–AACCTGGTTGATCCTGCCAG–3′ and 1528R 5′–TGATCCTTCTGCAGGTTCACCTAC–3′. The PCR was conducted using a final volume of 25 μL containing 3.5 mM of MgCl_2_, 0.5 mM of each primer, 0.2 mM of dNTPs, 0.6 units (U) of *Thermus aquaticus* (Taq) polymerase in 1× PCR buffer, 0.1 μg of extracted parasite genomic DNA, and nuclease-free sterile double-distilled water up to 25 μL. The thermocycling conditions were as follows: 94°C for 2 minutes; 3 cycles of 94°C for 40 seconds, 51°C for 40 seconds, 72°C for 1 minute; 5 ‘touchdown’ cycles of 94°C for 40 seconds, 50°C–46°C for 40 seconds (dropping 1°C per cycle), 72°C for 1 minute; 35 cycles of 94°C for 40 seconds, 45°C for 40 seconds, 72°C for 1 minute; and a final extension at 72°C for 5 minutes. DNA gel electrophoresis (1.5% agarose gel) was used to confirm the amplified product (10–15 μL). The DNA bands were stained with ethidium bromide (0.5 μg/mL) against the GeneRuler 100 bp Plus ready-to-use DNA ladder (Fermentas, Waltham, MA, USA) as a molecular weight marker. A DNA gel purification kit (Abgene, Portsmouth, NH, USA) was used to purify the appropriate-sized PCR amplicons from the gel. The sequencing reactions were carried out with 10 µL and contained 1 µL BigDye Terminator (BDT) v3.1 (Applied Biosystems, Waltham, MA, USA), 2 µL of BDT buffer, 0.16 µM of primer, and 1–2 µL of PCR product. Sequencing products were purified with the DyeEx® 2.0 Spin Kit (Qiagen) and run on a 3130*xl*Genetic Analyzer (Applied Biosystems). The sequences were aligned and compared with different trypanorhychid species previously accessed in GenBank.

### Phylogeny

Phylogenetic analysis and evolutionary history for the isolated parasites were carried out using the Maximum Likelihood method and Tamura 3-parameter model. The recovered sequences were aligned and compared against Trypanorhyncha species previously accessible in the GeneBank. Sequence identity for the recovered data was checked using the Basic Local Alignment Search Tool (BLAST, available at http://blast.ncbi.nlm.nih.gov/Blast.cgi). The sequence trimming for the congeneric species recovered was carried out by BIOEDIT v7.5.3; sequence alignment was done by CLUSTAL W v2 while the phylogenetic tree was constructed using MEGA 7 programme.

## Results

**Figure 1:**
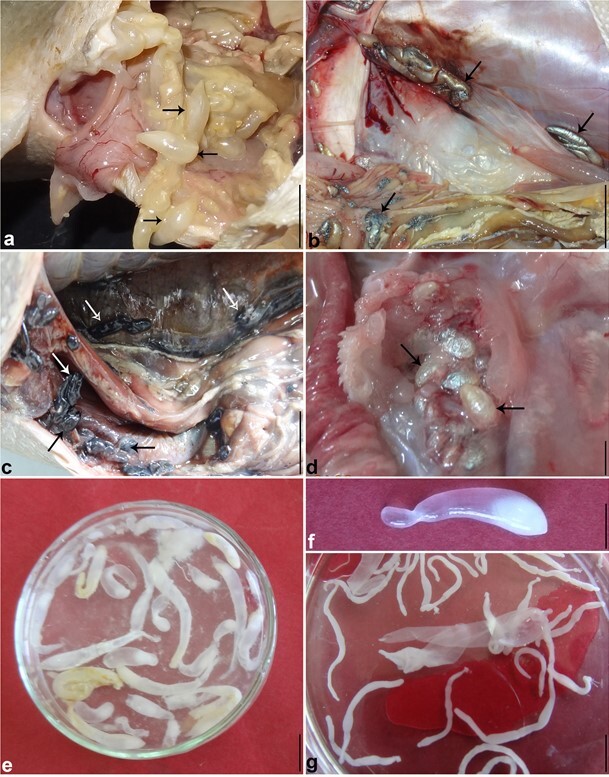
**(a-d)** Photographs showing encapsulated blastocysts of trypanorhynch metacestodes (arrows) in the peritoneal cavity of: (**a)** *Seriola dumerili***; (b)** *Pseudocarans dentex*; **c.** *Epinephelus haifensis*; (**d)** *Mycteroperca rubra*; (**e, f)** Isolated blastocysts; (**g)** Generated post larvae (plerocerci), Bars: a-d 1 cm; e-f 0.5 cm.

Four species of trypanorhynch cestodes were isolated from the peritoneal cavity of the examined fish. All of the included species represent the first locality records in the investigated area. These included *Callitetrarhynchus gracilis* (Figure 1a) isolated from the greater amberjack *Seriola dumerili* (46.7%, 7/15), *Callitetrarhynchus speciosus* (Figure 1b) from the gulley jack *Pseudocarans dentex* (50.0%, 10/20), *Protogrillotia zerbiae* (Figure 1c) from the Haifa grouper *Epinephelus haifensis* (76.5%, 13/17), and *Grillotia brayi* (Figure 1d) from the mottled grouper *Mycteroperca rubra* (65.2%, 15/23). The majority of trypanorhynch cestodes were found in the body cavity and mesenteries. Worms were encapsulated within whitish blastocysts (Figure 1e, f); after rupture, each blastocysts generated a post larva called plerocercus (Figure 1g; *plural* plerocerci).

### Morphology

Family: Lacistorhynchidae (Guiart, 1927)

Genus: *Callitetrarhynchus* (Pintner, 1931)

*Callitetrarhynchus gracilis* (Rudolphi, 1819)

**Figure 2:**
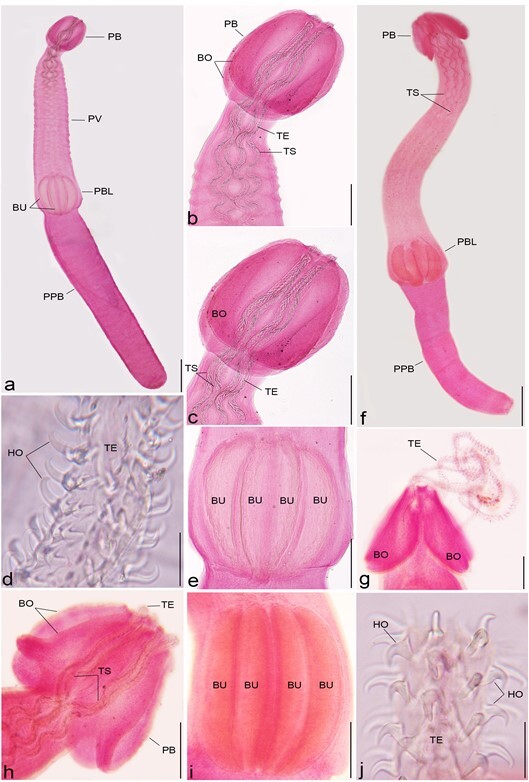
Photomicrographs of trypanorhynch metacestodes, carmine stained isolated from the examined fish showing: (**a-e)** *Callitetrarhynchus gracilis*, **(a)** Entire worm, lateral view, PB pars bothridialis, PV pars vaginalis, PBL pars bulbulosa, PPB pars post bulbulosa, BU bulbs, Bar 500 µm; (**b, c)** The anterior part, BO bothridia, TS tentacle sheaths, TE tentacles, Bar 200 µm; (**d)** Tentacle (TE) and hooks (HO), Bar 40 µm; **(e)** Four bulbs (BU), Bar 200 µm; (**f-j)**. *Callitetrarhynchus speciosus*, **(f)** Entire worm, lateral view, Bar 500 µm; **(g, h)** the anterior part, BO bothridia, TS tentacle sheaths, TE tentacles, Bar 200 µm; **(i)** Four bulbs, Bar 200 µm; **(j)** Tentacles (TE) and hooks (HO) Bar 20 µm.

Description (based on 10 plerocerci): The host capsule ranged from bladder–like to elongate and was usually white; blastocyst 17–35 (27.6) mm long. The post larva had an elongated body (Figure 2a), 9.2–16.8 (14.44) mm long × 0.71–0.95 (0.82) mm wide with elongated scolex (Figure 2 b, c, 4a) measured 6.7–9.72 (7.8) mm long and featuring two short, heart-shaped bothridia and a long tail. Bothridia 0.95–1.8 (1.25) mm long x 0.33–0.81 (0.50) mm wide; and the length of the pars vaginalis was 1.7–4.3 (3.0) mm long, that of the pars bulbosa was 0.75–0.93 (0.88) mm, and that of the pars post bulbosa was 0.20–0.41 (0.21) mm. The tentacles (Figure 2 d, 4b) were elongated and tapered, without basal swellings or a ring of larger hooks, and the tentacle sheaths were tightly coiled. The tentacle bulbs (Figure 2e) reached the end of the scolex, but they did not occupy its entire width; they were about three times longer than their width. The distinct basal armature consisted of rows of uncinate hooks continued by hooks of different shapes and sizes. The metabasal armature was poeciloacanthous and heteromorphous, and began on the internal surface. Hooks 1 (1´) uncinate, hooks 2 (2´) long and uncinate, hooks 3 (3´) large and falciform with large bases, hooks 4 (4´) and 5 (5´) falciform, hooks 6 (6´) spiniform and arranged on the external surface, hooks 7 (7’) large, and hooks 8 (8’) smaller, where both were uncinate and slender.

**Figure 4:**
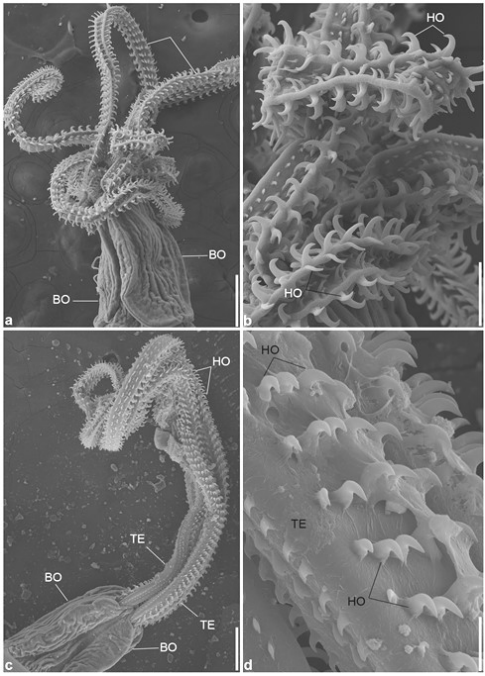
Scanning electron micrographs of a plerocercoid of: (**a, b**) *Callitetrarhynchus gracilis*, (**c, d**) *Callitetrarhynchus speciosus*, (**a**) Pars bothridialis of *C. gracilis,* BO bothridia, TE tentacles, Bar 200 µm; (**b**) Enlarged tentacle (TE) with hooks (HO), Bar 50 µm; (**c**) Pars bothridialis of *C. speciosus*, BO bothridia, TE tentacles, Bar 200 µm; (**d**) Enlarged of tentacle (TE) with hooks (HO), Bar 20 µm.

### Taxonomic summary

Host: the greater amberjack *Seriola dumerili* (Family: Carangidae).

Locality: coasts of Alexandria along the Mediterranean Sea, Egypt.

Infection site: body cavity and mesenteries as encapsulated larvae.

Prevalence: seven fish out of 17 (46.7%) were naturally infected.

Voucher material: three stained slides as whole mount (ZOO. BIO21.1–3) in addition to 70% ethanol preserved samples in vials are deposited in the parasites section, Zoology department, Faculty of Science, Cairo University, Egypt.

*Callitetrarhynchus speciosus* (Linton, 1897)

Description (based on 8 plerocerci): Blastocysts white in color and measured 4–10 (7.5) mm long. The post larva (Figure 2f) had an elongated, thin, and acraspedote scolex measuring 5.5–9.92 (7.7) mm long. Two bothridia with no or weak notched posterior margins (Figure 2 g, h, 4c) measured 0.86–1.4 (1.10) mm and 0.30–0.71 (0.50) mm wide. Pars vaginalis 1.2–4.5 (3.3) mm long; the tentacle sheaths were regularly sinuous and enlarged anteriorly, but were less sinuous on the pars botrialis region. Bulbs elongated (Figure 2i). Pars post bulbosa 0.20–0.32 (0.28) mm in length. The metabasal armature are heteromorphous and poeciloacanthous with hollow hooks arranged spirally from the internal surface (Figure 2 j, 4d). Hooks 1 (1´) and 2 (2´) uncinate and long; hooks 3 (3´), 4 (4´), and 5 (5´) falciform; hooks 6 (6´) spiniform and located near the external surface; and satellite hooks 7 (7') and 8 (8') were of the same size and a slender uncinate shape.

### Taxonomic summary

Host: the gulley jack *Pseudocarans dentex* (Family: Carangidae).

Locality: coasts of Alexandria along the Mediterranean Sea, Egypt.

Infection site: body cavity and mesenteries as encapsulated larvae.

Prevalence: ten fish out of 20 (50%) were naturally infected.

Voucher material: five stained slides as whole mount (ZOO. BIO21.4–8) in addition to 70% ethanol preserved samples in vials are deposited in the parasites section, Zoology department, Faculty of Science, Cairo University, Egypt.

Genus: *Protogrillotia* (Palm, 2004)

*Protogrillotia zerbiae* (Palm, 2004)

**Figure 3:**
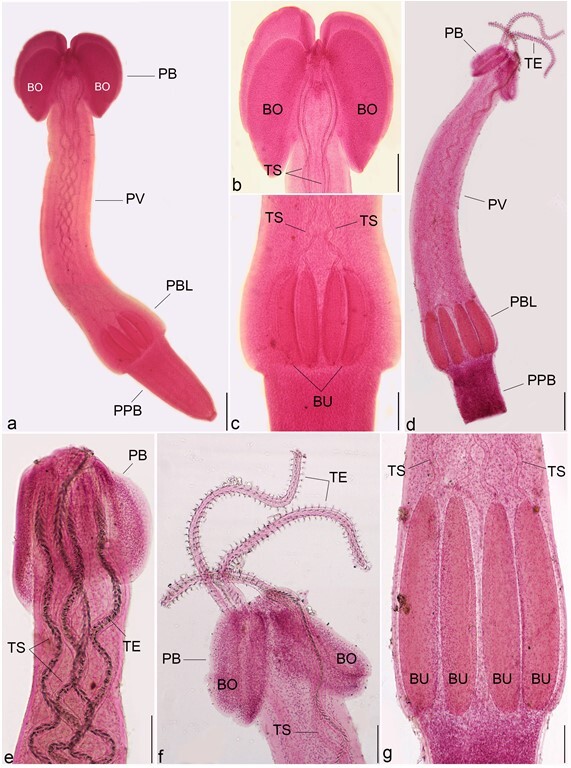
Photomicrographs of trypanorhynch metacestodes, carmine stained isolated from the examined fish showing: (**a-c)** *Protogrillotia zerbiae*, **(a)** Entire worm, lateral view, PB pars bothridialis, PV pars vaginalis, PBL pars bulbulosa, PPB pars post bulbulosa, BU bulbs, Bar 200 µm; **(b)** The anterior part, BO bothridia, Bar 100 µm; **(c)** Four bulbs (BU), Bar 100 µm; (**d-g).** *Grillotia brayi*, **(d)** Entire worm, lateral view, Bar 200 µm; **(e, f)** The anterior part, BO bothridia, TS tentacle sheaths, TE tentacles, Bar 100 µm; **(g)** Four bulbs, Bar 100 µm.

Description (based on 8 plerocerci): The scolex long (Figure 3a), slender, and craspedote, measuring 3.23–4.56 mm long × 0.21–0.40 mm wide at the pars bothriallis, 0.40–0.69 mm at the pars vaginalis, and 0.33–0.46 mm at the pars bulbosa. There were two bothria (Figure 3b) that were patelliform and posteriorly notched with a prominent rim. The length of the pars bothridialis was 1.63–1.95 mm, that of the pars vaginalis was 0.75–0.96 mm, that of the pars bulbosa was 0.22–0.35 mm, and that of the pars post bulbosa was 0.11–0.22 mm. Bulbs ovoid and elongated (Figure 3c), measuring 0.27–0.38 mm long × 0.30–0.49 mm wide. Tentacle sheaths highly coiled and 27–28 µm long; the tentacles reached the apical end of the bulbs with no tentacular swelling. The tentacular armature was heteroacanthous and heteromorphous. Hooks were arranged in ascending rows of seven enlarged principal hooks. Hooks 1–6 (1′–6′) uncinate, hooks 7 (7′) slender with a short base and slightly uncinate, and hooks 7 (7′) were spiniform hooks.

### Taxonomic summary

Host: the Haifa grouper *Epinephelus haifensis* (Family: Serranidae).

Locality: coasts of Alexandria along the Mediterranean Sea, Egypt.

Infection site: body cavity and mesenteries as encapsulated larvae.

Prevalence: thirteen fish out of 17 (76.5%) were naturally infected.

Voucher material: three stained slides as whole mount (ZOO. BIO21.9–11) in addition to 70% ethanol preserved samples in vials are deposited in the parasites section, Zoology department, Faculty of Science, Cairo University, Egypt.

Genus: *Grillotia* (Guiart, 1927)

*Grillotia brayi* (Beveridge & Campbell, 2007)

**Figure 5:**
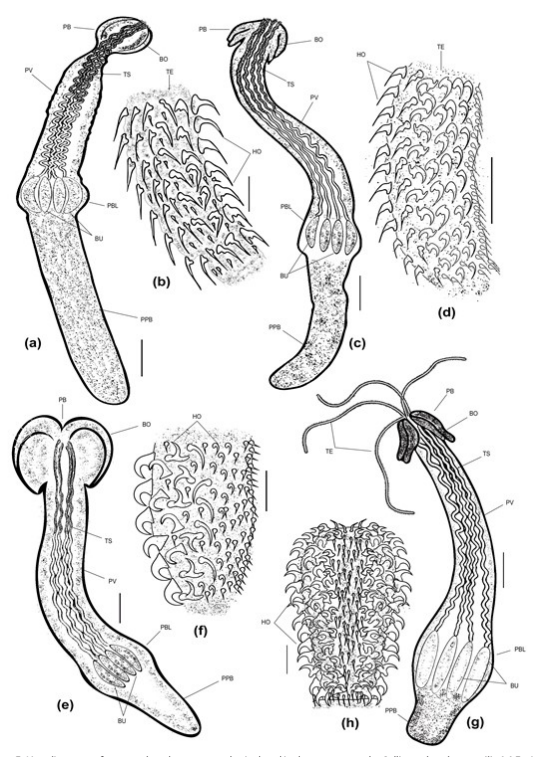
Line diagrams of trypanorhynch metacestodes isolated in the present study: *Callitetrarhynchus gracilis*, **(a)** Entire worm, Bar 500 µm; **(b)** Enlarged tentacle, Bar 20 µm; *Callitetrarhynchus speciosus,* **(c)** Entire worm, Bar 500 µm; **(d)** Enlarged tentacle, Bar 20 µm; *Protogrillotia zerbiae*, **(e)** Entire worm, Bar 200 µm; **(f)** Enlarged tentacle, *Grillotia brayi*, **(g)** Entire worm, Bar 200 µm; **(h)** Enlarged tentacle, Bar 50 µm. PB pars bothridialis, PV pars vaginalis, PBL pars bulbulosa, PPB pars post bulbulosa, BO bothridia, TS tentacle sheaths, TE tentacles, HO hooks, BU bulbs.

Description (based on 7 plerocerci): The scolex of the isolated plerocerci were acraspedote 4.27–9.24 (6.65) mm long × 0.70–1.30 (1.12) mm wide (Figure 3d). Pars bothrialis 0.71–1.42 (1.01) mm long, with two large sub–cordiform bothria 0.81–1.30 (1.02) mm in length (Figure 3e, f). Pars vaginalis 2.21–3.20 (2.45) mm long, the elongated bulbs were 1.75–2.82 (2.16) mm long and 0.16–0.40 (0.21) mm wide (Figure 3g), prebulbar organ absent. Pars post-bulbosa short at 0.20–0.51 (0.39) mm long. The tentacles did not feature basal swellings; the sheaths were highly coiled and sinuous. The armature was heteroacanthous and heteromorphous. The hooks began on the internal surface of the tentacle and were uncinate, 7–15 (13) mm long, while the base was 4–10 (7) mm. The principal rows of the metabasal region were comprised of 4 hooks with a sub–triangular broad base and they were posteriorly directed with a curved, slender, aciculate blade. The total number of intercalary hooks was 10–12. Line diagrams for the recovered plerocerci and their tentacles armature were shown in Figure 5.

### Taxonomic summary

Host: the mottled grouper *Mycteroperca rubra* (Family: Serranidae).

Locality: coasts of Alexandria along the Mediterranean Sea, Egypt.

Infection site: body cavity and mesenteries as encapsulated larvae.

Prevalence: fifteen fish out of 23 (65.2%) were naturally infected.

Voucher material: three stained slides as whole mount (ZOO. BIO21.12–14) in addition to 70% ethanol preserved samples in vials are deposited in the parasites section, Zoology department, Faculty of Science, Cairo University, Egypt.

### Molecular study

**Figure 6:**
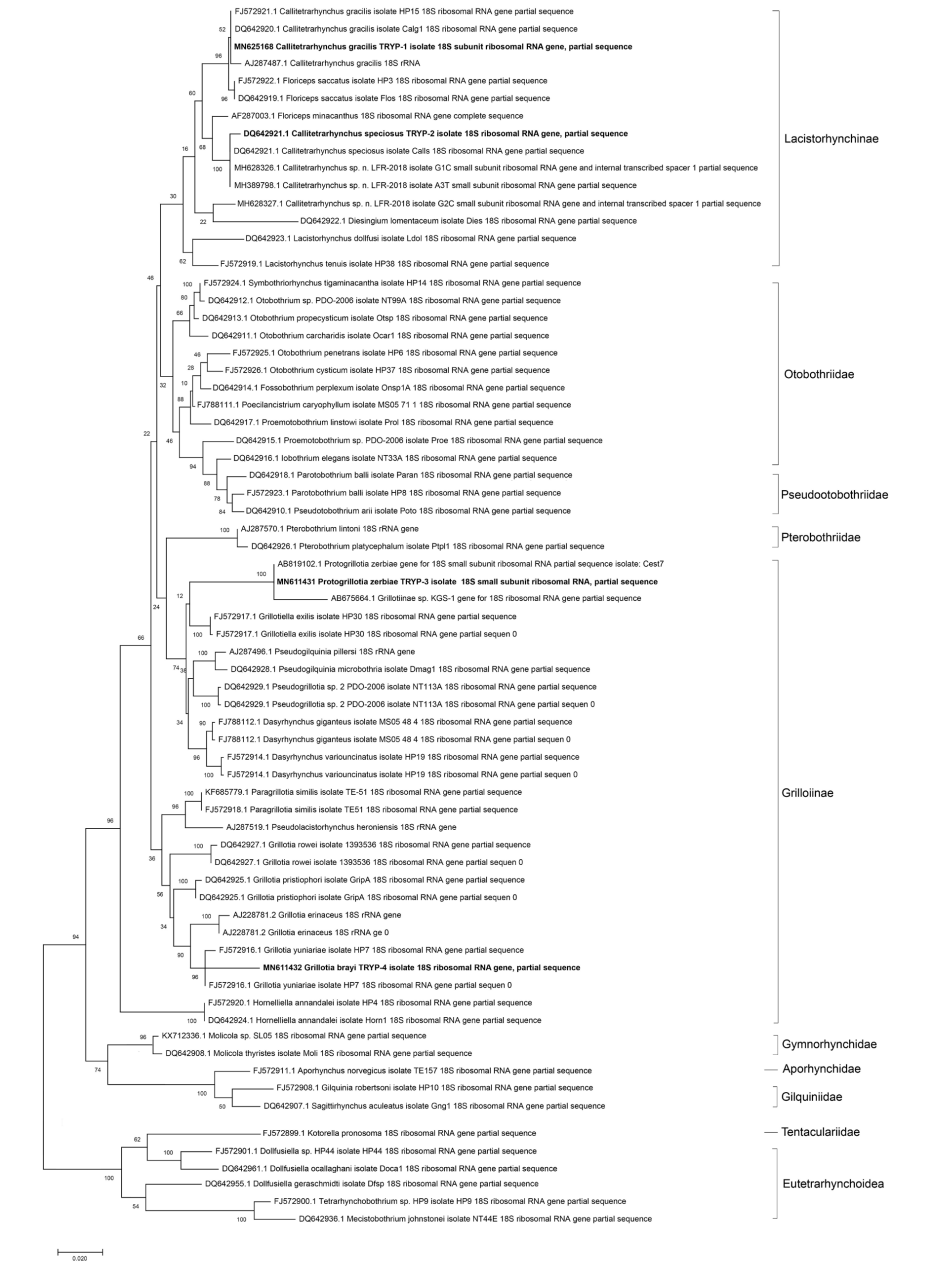
Phylogenetic analysis and evolutionary history using the Maximum Likelihood method and Tamura 3-parameter model according to the parasites 18s rRNA sequence analysis, the percentage of trees in which the associated taxa clustered together is shown next to the branches. Initial tree (s) for the heuristic search was obtained by applying the BioNJ method to a matrix of pairwise distances estimated using the Maximum Composite Likelihood (MCL) approach. The tree is drawn to scale, with branch lengths measured in the number of substitutions per site. This analysis involved 69 nucleotide sequences. There were a total of 1094 positions in the final dataset.

According to the phylogenetic analyses (Figure 6), there are two major lineages within the order Trypanorhyncha: the first clade includes the superfamilies “Eutetrarhynchoidea” and “Tentacularioidea”. Members of the second major lineage include monophyletic trypanorhych cestodes. Families Gymnorhynchidae, Aporhynchidae, and Gilquiniidae are the sister groups to this clade. The monophyletic clade of Lacistorhnchinae has a sister group that includes members of the family Otobothriidae. Pseudootobothriidae includes members that are sister to Otobothriidae, the tree also supports that three sister groups are within Otobothriidae and that one group encompasses Proemotobothrium, Iobothrium and Pseudootobothriidae. The constructed tree was polyphyletic and included the four queued species in different clades. The query sequences of the cestode parasite isolated from *Seriola dumerili* showed different identities from *C. gracilis*, which was identified in GenBank. The maximum identity was 94.24% (Acc. No. MG693781.1), followed by 91.12% (FJ572921.1, DQ642920.1), and 91.08% (Acc. No. LC037194.1); it was deposited in GenBank under accession number MN625168. While the 18s RNA sequences of the parasite isolated from *Pseudocarans dentex* yielded an identity percentage of 97.47% with 18s ribosomal RNA sequences of *C. speciosus* recovered from GenBank (accession number: DQ642921.1). The recovered sequences were deposited in GenBank under accession number MN625169. The BLAST results also indicated that the RNA sequences of the cestode isolated from *Epinephelus haifensis* showed high similarity (identity percentage of 95.55%) with the previously deposited sequences of *Protogrillotia zerbiae* in GenBank (AB819102.1, AB819099.1, AB819101.1). The recovered sequences were deposited in GenBank under accession number MN611431. The 18 RNA sequences of the parasite recovered from *Mycteroperca rubra* showed BLAST similarities with some species of the genus *Grillotia*, with a maximum identity percentage of 89.04% with *Grillotia yuniariae* (FJ572916.1), 87.53% with *Grillotia pristiophori* (DQ642925.1), 87.41% with *Grillotia erinaceus* (AJ228781.2), and 86.90% with *Grillotia rowei* (DQ642927.1), which supported the inclusion of query sequences within the genus *Grillotia*. However, it was identified as a different species given the low identity percentage. The recovered sequences of this parasite were deposited in GenBank under accession number MN611432.

## Discussion

There are 277 species of marine cestodes within Trypanorhyncha Diesing, 1863 that use elasmobranches as their final hosts (Palm, 2004; Palm et al., 2009). The present study provides the first data on the spectrum of trypanorhynch infestations among commercially important teleost fishes from the Mediterranean Sea, as illustrated through morphological and molecular analyses. The four recovered metacestodes in the present study possess most of the characteristic features of the order Trypanorhyncha, which include the following: the presence of two or four bothria and a tentacular apparatus with 4 eversible tentacles at its apex; and tentacles that generally bear a complex array of diverse hooks used to attach to the mucosa of the gastrointestinal tract (Dollfus, 1942; Richmond & Caira, 1991; Campbell & Beveridge, 1994; Palm, 1995, 1997; Morsy et al., 2013). Cestodes in this group are unique because a specialist can often identify the larva species, usually in an invertebrate or teleost intermediate host, simply by observing the morphology of the scolex. The cestodes serve as a model group for understanding the patterns of host specificity (Palm & Caira, 2008), zoogeographic distribution, and parasite evolution within the marine ecosystem (Palm, 2004; Palm & Klimpel, 2007; Palm et al., 2007; Palm et al., 2009; Palm & Caira, 2008). The recovered *C*. *gracilis* and *C. speciosus* possessed a characteristic morphology similar to those of previously described trypanorynch cestodes of the genus *Callitetrarhynchus* (Dollfus, 1942; Carvajal & Rego, 1985; Palm, 2004). There are many species of *Callitetrarhynchus* that were previously recorded from perciform fish teleosts of the families Scombridae, Lutjanidae, and Serranidae (Palm, 2004). *C*. *gracilis* and *C*. *speciosus* plerocerci recorded in this work are similar to species that parasitize marine fish worldwide. Palm (2004) mentioned that these two species varied greatly in size between different hosts. This may be related to the concept that the small form of *C*. *gracilis* plerocercus infects small fish species (such as cluppeids), while the large form infects large fish species (such as scombrids). The morphology of *C. speciouses* plerocercei isolated in the current study resembles that of the species that infect sciaenid fish of the *Epinephelus* species reported previously by Lima (2004); they are similar in terms of their morphology of the pars post bulbosa and the presence of a spiral arrangement of unicate hooks. Also, similar plerocercei were recorded by Pereira & Boeger (2005) from *Micropogonias furnieri* and *Cynoscion guatucupa* from Brazil; they differed from the recorded parasite in terms of tentacle hook shape, which is heteromorphous and heterocanthus. *Grillotia brayi* closely resembles *G. borealis*, *G. dollfusi*, and *G. musculara* in the absence of specialized hooks at the base of the tentacle (Santoro et al., 2020). It differs from *G. dollfusi* in that it has a much longer pars vaginalis, an attenuated anterior part of the bulb, and smaller hooks in the principal row. *G. brayi* differs from *G. musculara* as the former has uncinate rather than spiniform hooks arranged at the external surface of the tentacle. It also differs from *G. Borealis* given the absence of a bifid tip on the hooks; the external surface of the tentacles possesses hooks that extend to its base with no areas free of hooks, as in *G. Borealis* and *G. dollfusi*. *Protogrillotia zerbiae* recovered in the present study is morphologically similar to the cestode isolated previously from cultured and wild amberjacks *Seriola dumerili* and *Seriola rivoliana*. Tamaru et al. (2016) conducted a parasitological survey on “kahala” *Seriola dumerili* caught in Hawaii, they found blastocysts in the muscle of the head and along the back, just below the dorsal fin, in 20 out of 23 fish examined. The intensity of infection ranged from 1**–**7. Based on morphology, they assigned the cestode to the genus *Protogrillotia*. Palm (1995) proposed a new species, *Pseudogrillotia zerbiae*, for the plerocercus of the greater amberjack collected from the musculature of *Seriola dumerili* in Ocean Springs, Mississippi, USA, and synonymized the Hawaiian cestode with this new species. Later, Palm (2004) included *P. zerbiae* into a new genus, *Protogrillotia*, and thus *Pseudogrillotia zerbiae* was renamed to *Protogrillotia zerbiae*. The diagnosis of and differentiation between *Protogrillotia* and *Grillotia* Guiart (1927) are not clearly understood. Beveridge et al. (1999) studied the phylogeny of some species of Trypanorhyncha, with no records of Plerocerci, and the blastocysts differed from those identified in the present study, which isolated Plerocerci and blastocysts of *P. dollfusi*. The authors did not develop a protocol to differentiate between *Protogrillotia* and *Grillotia*. The species recorded in the current study are similar to species from the order Lacistorhynchoidea. The phylogenetic analysis used 18s small ribosomal RNA for the recovered metacestodes, which led to the construction of multiple alignments that supported the taxonomic position of these parasites representing three genera: *Callitetrarhynchus*, *Protogrillotia*, and *Grillotia*. These genera are sister taxons to *Floriceps saccatus*, *Grillotiinae* sp., and *Hornelliella annandalei*, respectively, in accordance with Olson et al. (2010). The molecular evidence shows that Trypanorhincha consists of two well-supported lineages, and important morphological cross-linking has been mapped, where the highly variable armature pattern represents the main morphological diagnostic tool. The molecular phylogeny and tree topology in the present study are similar to the cladistic analysis of trypanorhynch cestodes reported by Palm (2004), where trypanorhynch cestodes split into two main clades: the first constitutes members of the superfamily Eutetrarhynchoidea, Tentacularioidea, while the second mainly includes Grilloiinae, Lacistorhynchinea, and Otobothrioidea. The branch including lacistorhynchoids consists of two main paraphyletic clades: poeciloacanthous multiatypical (*Dasyrhynchus*, *Protogrillotia*, and *Grillotia*) and poeciloacanthous atypical (*Callitetrarhynchus*). This clade has a monophyletic sister taxon, Otobothrioidea (Palm & Overstreet, 2000; Palm et al., 2009).

## Conclusion

Both the molecular analysis and morphological characterization performed in the present study support the taxonomic identification of four parasitic metacestodes: *C. gracilis*, *C. speciosus*, *P. zerbiae*, and *G. brayi*. To ensure good food hygiene, trypanorhynch cestodes should be removed from infected fish, as parasitized fish are generally rejected by consumers due to their repulsive appearance, and humans are at greater risk for accidental infection and allergic reactions following the ingestion of raw infected fish meat.
